# Dr. James N. Riley

**Published:** 1917-11

**Authors:** 


**Dr. James N. Riley,** Atlanta Medical College 1857, a Confederate surgeon, died at his home in Stamping Ground, Ky., Sept. 18, aged 90.

